# Does Length of Time Caregiving Affect Net Worth? Caregivers Approaching Retirement Age

**DOI:** 10.1093/geroni/igaa057.098

**Published:** 2020-12-16

**Authors:** Shirley Porterfield, Linda Quinn, Patricia Stoddard Dare, LeaAnne DeRigne, Miyuki Fukushima Tedor, Cyleste Collins

**Affiliations:** 1 University of Missouri–St. Louis, St Louis, Missouri, United States; 2 Cleveland State University, Cleveland, Ohio, United States; 3 Florida Atlantic University, Boca Raton, Florida, United States

## Abstract

Using a nationally representative sample of N=3,659 adults (mean age 55.6 in 2016) from the 2008, 2012, and 2016 rounds of the National Longitudinal Survey of Youth 1979, we examine the impact of work-limiting health conditions, caregiving, and the length of time spent in a caregiving role inside and outside the home on total family net worth. Repeated measures analysis finds having a chronically ill or disabled household member (CIOD), or caring for a household member with a CIOD inside the home was associated with reduced total family net worth (TFNW; -$146.7, p<0.002), on average, across the three time periods. In-home caregiving across time periods does not have an additive effect, but rather appears to result in a one-time drop in TFNW. Respondents who provide care for out-of-home family or friends report higher TFNW on average over time ($73.3K, p<0.001). Respondents with a work-limiting health condition report a lower mean TFNW over this time span (-$53.1K, p<0.005). Caregiving inside the home has 2-3 times the impact on TFNW as having a work-limiting health condition, though in the overall model, the effect of the two variables together on TFNW is additive. An adult is at the greatest disadvantage with respect to financial preparedness for retirement if they are both a caregiver (or have a CIOD in their household) and have a work-limiting health condition than if they have only one of these characteristics or have neither characteristic. Even a single time period of caregiving reduces total family net worth over time.

